# PD-L1 Distribution and Perspective for Cancer Immunotherapy—Blockade, Knockdown, or Inhibition

**DOI:** 10.3389/fimmu.2019.02022

**Published:** 2019-08-27

**Authors:** Yilun Wu, Weiyu Chen, Zhi Ping Xu, Wenyi Gu

**Affiliations:** Australian Institute for Bioengineering and Nanotechnology, The University of Queensland, St. Lucia, QLD, Australia

**Keywords:** cancer immunotherapy, PD-1/PD-L1 immune checkpoint, cellular PD-L1 distribution, gene silencing, PD-L1 regulation, signaling pathway inhibitor, combination therapy

## Abstract

Cancer immunotherapy involves blocking the interactions between the PD-1/PD-L1 immune checkpoints with antibodies. This has shown unprecedented positive outcomes in clinics. Particularly, the PD-L1 antibody therapy has shown the efficiency in blocking membrane PD-L1 and efficacy in treating some advanced carcinoma. However, this therapy has limited effects on many solid tumors, suspecting to be relevant to PD-L1 located in other cellular compartments, where they play additional roles and are associated with poor prognosis. In this review, we highlight the advances of 3 current strategies on PD-1/PD-L1 based immunotherapy, summarize cellular distribution of PD-L1, and review the versatile functions of intracellular PD-L1. The intracellular distribution and function of PD-L1 may indicate why not all antibody blockade is able to fully stop PD-L1 biological functions and effectively inhibit tumor growth. In this regard, gene silencing may have advantages over antibody blockade on suppression of PD-L1 sources and functions. Apart from cancer cells, PD-L1 silencing on host immune cells such as APC and DC can also enhance T cell immunity, leading to tumor clearance. Moreover, the molecular regulation of PD-L1 expression in cells is being elucidated, which helps identify potential therapeutic molecules to target PD-L1 production and improve clinical outcomes. Based on our understandings of PD-L1 distribution, regulation, and function, we prospect that the more effective PD-L1-based cancer immunotherapy will be combination therapies.

## Introduction

Cancer immunotherapy is a specific method to eliminate cancer cells by enhancing or modulating the host immune system. The immune checkpoint molecules regulate the immune balance, and the neutralization of immunosuppressive checkpoints can lead to cancer elimination. Among these immune checkpoints, the blockade of programmed death-protein 1 (PD-1) and its ligands 1 and 2 (PD-L1/2), an intrinsic negative checkpoint, leads to one of the most successful immunotherapies by enhancing T cell immune responses against tumor cells. Currently, the blockade of PD-1/PD-L1 can be achieved via three methods: (1) antibody blockade, (2) gene silencing, and (3) small-molecule pathway inhibition. The commercial PD-L1 antibodies have shown tremendous success, in particular, for advanced cancers such as melanoma and non-small lung cancers (1, 2). Out of the three methods the gene silencing strategy is less studied but has now attracted more attention due to the approach of inhibiting PD-L1 pathways.

The antibody-based treatment has shown in studies to be insufficient in all PD-L1 low expression cases, and even some PD-L1 overexpression cohort (1). Moreover, the overall response rate in most solid tumors is only around 20%. This deficiency suggests that deeper understanding of the PD-L1 mechanism is required. In addition, the cost of production and delivery, storage stability, and immunogenicity are also issues for the antibodies (3). Mechanically, the suppression of PD-L1 using gene silencing may be more efficient than antibody blockade, as a single interfering gene fragment is able to “switch off” the protein synthesis. This method is still regarded as a backup regimen for PD-L1 therapy due to the lack of commercialized products and other issues of gene/drug delivery. Other than direct PD-L1 gene silencing, the siRNAs can also benefit PD-L1 based treatment through regulating the involved expression signaling pathways, which can also be achieved by commercialized chemical inhibitors as well (4). These chemical inhibitors, with definite chemical structures, can offer benefits in terms of pharmacokinetics, druggability, and cost control. The drawback of this method is that the regulation downregulates PD-L1 molecules indirectly, resulting in a possible increase in time require for the downregulating pathway signal molecules to communicate to the PD-L1 molecules. In addition, small molecules of pathway inhibitors may induce drug resistance (4).

Recent studies have revealed that the PD-L1 molecules have a broad distribution in and outside cells. PD-L1 can be located extracellularly, intracellularly, and on the cell membrane. Elucidating the functions of PD-L1 at different locations and its transport could lead to deeper understanding of these treatment strategies, thus guide the choice of therapeutic approach. However, both the PD-L1 distribution and subsequent selection of a proper therapeutic regimen have not been clearly discussed. Herein, we have firstly summarized the current studies on PD-L1 distribution in this review article. Subsequently, the PD-L1 based immunotherapies in relation to these three methods are reviewed and compared. We have then provided our opinions regarding how to choose a personalized strategy for more effective PD-L1 based cancer therapy in the final prospective section.

## Background of PD-1/PD-L1 Based Cancer Immunotherapy

As one of the major threats to public health worldwide, cancer is responsible for millions of deaths annually, with a high morbidity (5). Simultaneously, trillions of dollars spent in cancer treatment further intensify the pressure upon our society and patient families (6). Since the last decade, the immunotherapy has become an efficient cancer treatment. With the onset of tumor, multiple immune resistance mechanisms, such as local immune evasion, tolerance induction, and immune edition, are developed for tumor escape from immune surveillance (7, 8). Thus, immunotherapy strategies against cancers are proposed to stimulate the effectors and/or counteract inhibitory and suppressive mechanisms (9), including the regulation of immune cells (vaccine and T cell engineering), cytokines (ILs, IFNs, TGFs, TNFs, etc.) and immune checkpoints. Recent discovery of immunosuppressive checkpoints, such as CTLA-4 (cytotoxic T-lymphocyte-associated protein 4) and PD-1, provides a very successful regimen to cancer immunotherapy (10, 11), which has been awarded Nobel Prize in 2018.

**Figure 1:**
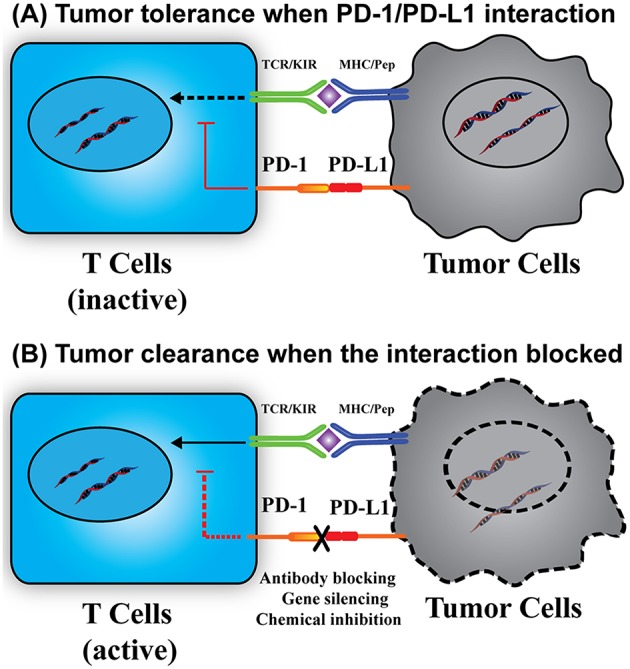
Immunotherapy based on PD-1/PD-L1 interaction. **(A)** The interaction of PD-1/PD-L1 causes tumor immune tolerance. The PD-1/PD-L1 interaction stimulates the downstream signals to suppress T cell activation, resulting in tumor cell survival. **(B)** Breakdown of the PD-1/PD-L1 interaction reactivates T cells and related immune responses. Without the PD-1/PD-L1 interaction, the suppression signal is removed, thus leading to T cell activation, proliferation, and cytokine generation and tumor cell elimination. KIR, killer-cell immunoglobulin-like receptor.

PD-L1, also known as CD274 and B7-H1, is a transmembrane protein commonly expressed on the surface of antigen presenting cells and tumor cells. PD-L1 specifically binds to its receptor, PD-1, which is expressed on the surface of immune-related lymphocytes, such as T cells, B cells, and myeloid cells (11, 12). In some solid or blood tumors, the PD-L1 can also bind to the PD-1 expressed on tumor cell surface (13–15). As shown in Figure 1, the binding of PD-L1 to PD-1 is able to activate the down-stream signaling of PD-1 receptor in T cells, thus inhibiting the proliferation, cytokine generation and release, and cytotoxicity of T cells. This down-regulation of immunity will prevent autoimmunity and chronic infection, many tumor cells also use this mechanism to protect themselves from immune attack, causing the so-called tumor immune evasion (12). PD-L1 mediated tumor immune resistance includes innate resistance caused by endogenously constitutive PD-L1 expression, and adaptive resistance caused by exogenously stimuli-inducible PD-L1 expression (16). Inhibition of either PD-1 or PD-L1 will enhance T cell responses to cancer. This approach is known as PD-1/PD-L1 based immunotherapy.

Based on this understanding, six antagonists have been developed and successfully approved by FDA. All of the approved antagonists are monoclonal antibodies, all have the ability to bind PD-1 or PD-L1. Notably, these antibodies demonstrate remarkably durable and persistent responses, with some patients remaining free from cancer progression for many years (17, 18). A brief summary of applicable cancer types with PD-L1 antibody response is illustrated in Table 1. Despite the success achieved in PD-1/PD-L1 antibody therapies, the objective response is not as high in PD-L1 positive cohort, and some unexpected responses occurred in PD-L1 negative cohort. Further studies of PD-L1 reveal its intracellular and extracellular existence, leading to the idea of whether antibody therapy is the optimal solution in all cancer cases.

**Table 1:** Applicable cancer types that respond to FDA proved PD-1/PD-L1 antibody products.

**FDA approved application**	**PD-L1 antibody**	**PD-1 antibody**
Urothelial carcinoma	Atezolizumab, Durvalumab	Nivolumab, Pembrolizumab
Non-small cell lung cancer (NSCLC)	Atezolizumab	Nivolumab, Pembrolizumab
Triple-negative breast cancer (TNBC)	Atezolizumab	
Small cell lung cancer (SCLC)	Atezolizumab	Nivolumab
Merkel cell carcinoma (MCC)	Avelumab	
Melanoma		Nivolumab, Pembrolizumab
Renal cell carcinoma (RCC)		Nivolumab
Hodgkin lymphoma (cHL)		Nivolumab, Pembrolizumab
Head and neck squamous cell cancer (HNSCC)		Nivolumab, Pembrolizumab
Gastric cancer		Pembrolizumab
Cervical cancer		Pembrolizumab
Microsatellite instability-high (MSI-H) or mismatch repair deficient (dMMR) metastatic colorectal cancer		Nivolumab, Pembrolizumab
Cutaneous squamous cell carcinoma (CSCC)		Cemiplimab

## PD-L1 Distribution and Function

### PD-L1 Formats

**Figure 2:**
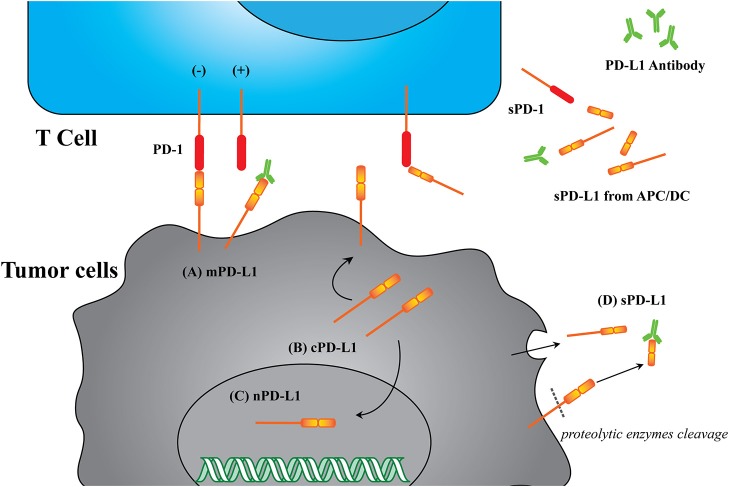
Illustration of different PD-L1 formats. **(A)** mPD-L1, located on the tumor cell membrane, is able to bind with PD-1 on T cells and response to tumor immune escape. PD-L1 antibody competitively binding to mPD-L1 breaks the tolerance, leading to tumor cell clearance. **(B)** cPD-L1 is located in cytoplasm, and potentiates to transfer to mPD-L1. **(C)** nPD-L1 is located in nuclei. Its aberrant upregulation is speculated to be associated with promoted cell chemo-resistance. **(D)** sPD-L1 refers to its soluble format in the serum, generated from either endogenous secretion or cleaved fraction of mPD-L1s. Both host cells (such as APC and DC) and tumor cells can be the source of sPD-L1. PD-L1 antibody therapeutic effect is limited to sPD-L1 consumption, and cannot modulate intracellular PD-L1.

Blocking of the cell surface protein PD-L1 is enough to enhance CTL cytotoxicity. Many investigations suggest that the broad distribution of PD-L1 in different cellular compartments can lead to deactivate of the CTLs (19). The known PD-L1 formats include membrane PD-L1 (mPD-L1) (20–22), cytoplasm PD-L1 (cPD-L1) (22, 23), nuclear PD-L1 (nPD-L1) (24, 25), and serum PD-L1 (sPD-L1). Meanwhile, the structures of these PD-L1 proteins are versatile, with some lacking transmembrane motifs and the potential of glycosylated modification or dimerization (19, 26, 27). Based on this information, we have herein proposed a new concept: the PD-L1 format will affect anticancer immunity. The PD-L1 format refers to its subcellular location and its structural integrity, which potentially affects its functions. The reported PD-L1 formats are summarized in Figure 2 and Table 2.

**Table 2:** The reported PD-L1 formats.

**Format**	**Location**	**Structure**	**Source**	**Possible functions**	**Treatment response**	Detection*	**References**
mPD-L1	Membrane	Integrity	Endogenous translation	Bind with PD-1 for immune regulation	Antibody, gene, and chemo-inhibitor	WB, IHC	(11, 12)
cPD-L1	Cytoplasm	–	Endogenous translation	Transfer to membrane, shorten disease-free survival, and cell growth and migration	Gene and chemo-inhibitor	WB, IHC	(23, 28)
nPD-L1	Nuclei	–	–	Enhance chemo-resistance	Gene and chemo-inhibitor	WB, IHC	(24, 25)
sPD-L1	Serum	Integrity or splice variant	Secretion from cancer cells/matured APCs	Bind with PD-1, associated with immune state	Antibody, gene, and chemo-inhibitor	ELISA	(29)
	Serum	Without transmembrane motifs	Enzyme cleavage	Bind with PD-1, associated with immune state	Antibody, gene, and chemo-inhibitor	ELISA	(19)
–	–	Dimeric	Crystallization	Functional units or evolution relic	–	–	(30)

* *The typical detection method: WB, Western blotting; IHC, Immunohistochemistry; ELISA, Enzyme-linked immunosorbent assay*.

As the major format, mPD-L1 holds the structure integrity and can bind to its receptor, PD-1, to modulate cancer cell immune tolerance, which can be reversed by utilizing a PD-L1 antibody. However, the antibody has limited influence on other intracellular PD-L1 formats (cPD-L1 and nPD-L1), which would affect the efficacy of PD-L1 based immunotherapy to some degree. Recent investigations are preferential to the blockade/suppression of mPD-L1 and extracellular sPD-L1, with lack of focus on other PD-L1 formats. Considering the roles of other PD-L1 formats in tumor immune resistance (Table 2), the improvement of these treatments may lie in how to deal with intracellular PD-L1 formats.

Due to the difference in the distribution and functions of these PD-L1 formats, how to deal with all of them needs to be considered and developed comprehensively in order to improve the PD-L1 based cancer immunotherapy.

### Cellular PD-L1 Distribution

Immunohistochemical (IHC) study of patient tumor tissues suggests that PD-L1 positive immune responses may appear on the tumor cell membrane (mPD-L1) and in the cytoplasm (cPD-L1). Given the transmembrane structure of PD-L1, the positive immune responses may partially be due to the obscure binding of antibody to specific domain of PD-L1 (31, 32). Meanwhile, cPD-L1, like other immune receptors such as CTLA-4, may be translocated onto the cell surface as the response to regulatory immune cells (33). In a study for papillary thyroid carcinoma, patients with positive cPD-L1 expression resulted in shorter disease-free survival than those with negative cPD-L1, highlighting the function of cPD-L1 (23).

The function of cPD-L1 may be related to the promotion of cancer cell growth. By gene silencing of PD-L1 with specific siRNA in SKOV3, an ovarian cell line with negative mPD-L1 but positive cPD-L1, Qu et al. demonstrated the inhibition in cell growth and migration (28). Given that the tumor intrinsic PD-L1 promotes MTORC1 signaling and inhibits autophagy, it is plausible to postulate that cPD-L1 protects cancer cells from death *via* the same pathway (34). Thus, knockdown of cPD-L1 with specific RNAs would benefit cancer immunotherapy. Similarly, a study in circulating tumor cells (CTCs) has showed that the nPD-L1 expression in patients is also significantly associated with a short survival (23).

Interestingly, chemotherapeutic drug treatments may induce expression of different PD-L1 formats. For example, doxorubicin treatment preferably increased expression of mPD-L1 and cPD-L1 in the nucleus, but suppressed the expression of mPD-L1 and cPD-L1 in the cytoplasm of MDA-MB-231 breast cancer cells. The aberrant expression of nPD-L1 is speculated to be associated with promoted cell chemo-resistance (25).

### Soluble PD-L1 in Serum

The soluble form of PD-L1 (sPD-L1) is often detected in sera/supernatants and its concentration is strongly associated with the expression level of PD-L1. Despite the obvious relation with mPD-L1, the generation of sPD-L1 is not clear. There are two possibilities: (1) the fragment from mPD-L1 cleaved by proteolytic enzymes, and (2) endogenous translated integrity protein or splice variant for secretion (19, 29, 35). Although the evidence suggests that it is only detectable in supernatants of mPD-L1+ cell lines with its concentration correlated with mPD-L1 expression to a certain extent. sPD-L1 is not always detected in all supernatants of mPD-L1+ cells. The investigation has also failed in utilizing sPD-L1 as a diagnostic biomarker in clear cell renal cell carcinoma, suggesting the resource of sPD-L1 is complicated. In addition, studies indicate the relationship of sPD-L1 and matured immune cells, whereas immature DCs, though express mPD-L1, do not have sPD-L1 in their supernatants. Moreover, the concentration of sPD-L1 significantly increases in the sera of aged health donors. Considering immunization potency decreases as age increases, it is plausible to postulate that the sPD-L1 concentration is correlated with human immune state (19, 26, 27). However, sPD-L1 binds with anti-PD-L1 antibody in circulation, suggesting that additional PD-L1 antibody may be required for the PD-L1 based cancer immunotherapy (19).

## PD-L1 Based Immunotherapy With Antibody Blockade

### Advances and Issues of PD-L1 Antibody Blockade

The PD-L1 antibody is able to bind with PD-L1 on tumor/antigen presenting cell surfaces, thus reversing the negative immune regulation. With great success in clinic trials, the development of PD-L1 antibodies has attracted wide attention. Up to now, three PD-L1 antibodies were approved by FDA, as listed in Table 3. Generally, the PD-L1 antibody treatment prolongs the survival (data not shown in the table) and generates the high objective response rate in the selected cohort (36–39). The highlight of this treatment lies in the relative low rate of high-grade treatment-related adverse events (tr-AE, judged as severe AE, grade ≥ 3). Compared to conventional therapies such as docetaxel treatment (severe tr-AE rate about 54%), the antibody treatments show a tremendous low tr-AE rate (40). Apart from solid tumors, PD-L1 antibodies respond very positively to blood cancers like leukemia and lymphoma (14, 15).

**Table 3:** Marketed PD-L1 antibodies.

**Name (Trade name)**	**Company**	**First FDA approval**	**Medical uses**	**ORR**		**tr-AE (Grade ≥3) (%)**
				**PD-L1+ (%)**	**PD-L1– (%)**	
Atezolizumab (Tecentriq)	Roche Genetech	2016	Urothelial carcinoma, NSCLC	26	9.5	17
Avelumab (Bavencio)	Merck Serono and Pfizer	2017	Merkel-cell carcinoma	53.8	4.2	6.8
Durvalumab (Imfinzi)	AstraZeneca	2017	Urothelial carcinoma	31	0	4.9

*ORR, objective responsive rate to solid tumor; AE, adverse event; tr-AE, treatment-related adverse event; NSCLC, non-small cell lung cancer*.

*Data source: highlights of prescribing information for Tecentriq, Bavencio, and Imfinzi. ORR and AE data refer to Apolo et al. (36), Massard et al. (37), McDermott et al. (38), Ning et al. (39)*.

Although PD-L1 based therapy could provide a specific and relative safe anti-cancer strategy, there are still several issues unsolved. The objective response (OR) to the treatment relies much on the expression of mPD-L1, showing an obvious correlation to PD-L1 positive cohort (Table 3). Even though, the outcome of PD-L1 antibody therapy cannot be fully predicted according to the expression of PD-L1 (41). More than half of patients with positive PD-L1 expression cannot benefit from the treatment. Very interestingly, there was fewer responses to PD-L1 antibody observed in the PD-L1 negative cohort, for uncertain reasons (42). The gene variation among individuals further increases this uncertainty and potential risk of PD-L1 antibody-based immunotherapy. The hyper-progressors of tumor have been observed in some patients with MDM2/MDM4 amplification (four of six patients) or EGFR aberrations (two of 10 patients) after anti-PD-1/PD-L1 mono-treatment (43). Moreover, the immune checkpoint-based treatment interferes the normal regulation of the immune system. Given this peculiar mechanism, the usage of PD-L1 antibodies brings immune-related adverse events (ir-AE) into safety consideration. A previous meta-analysis on patients with marketed PD-1 antibodies has underlined the increasing risk of pneumonitis in all-grade patients compared to chemotherapy and/or targeted drugs (44, 45). Similarly, the PD-L1 antibodies also show pneumonitis as the most severe ir-AE, with relative lower rates in all-grade and especially high-grade patients (refer to highlights of prescribing information of antibodies). According to the WHO database, patients with PD-1/PD-L1 antibody treatment also face the fatal risk of fulminant immune-related myocarditis (46, 47).

### The Influence of PD-L1 Distribution on the Therapy

Based on the description of PD-L1 distribution, the antibody blockade is theoretically only efficient for mPD-L1 and sPD-L1, with limited influence on intracellular PD-L1 formats. Considering the translocation of cellular PD-L1, the antibody influence on intracellular PD-L1 would be associated with the pharmacokinetics. With the blocking antibody eliminated by proteolytic degradation or systemic clearance, the intracellular PD-L1 may transfer to the cell surface and resume the ability for immune escape. Therefore, we reasonably postulate the intracellular PD-L1s are the reservoir for mPD-L1, which may explain some failure in PD-L1 positive cohort as this translocated mPD-L1 requires more frequent antibody administration and higher dosage for efficient cancer immunotherapy.

Despite that the positive PD-L1 expression is the premise of PD-L1 antibody treatment logically, it is not always in concordance to the PD-L1 positive and objective response in patients (Table 3). This phenomenon is believed to be associated with the complicated peripheral environment around the tumor tissue. With the understanding of PD-L1 distribution, we propose a new hypothesis that all PD-L1 formats, not only mPD-L1, are able to influence the response to the antibody. The intracellular PD-L1 formats may translocate to the membrane or secrete outside cells, and bind to antibodies, contributing to the ORR in PD-L1- cohort (Table 3). The mPD-L1, on the other hand, would translocate into cells as cPD-L1 under the stress of antibody, causing the low (<50%) ORR in PD-L1+ cohort (Table 3).

## Knockdown of PD-L1 Expression

The idea of using gene silencing strategy to “switch off” PD-L1 translation, or PD-L1 upregulation, may be a new therapy. Gene silencing uses a small interfering RNA (siRNA) to knockdown PD-L1 directly in tumor cells or uses microRNAs (miRs) to regulate epigenetically. The gene silencing strategy can also be used in PD-L1 modulation on host immune cells, leading to the enhancement of immune responses to cancers.

### Silencing PD-L1 in Tumor and Host Immune Cells

Downregulation of tumor PD-L1 expression, by utilizing PD-L1 siRNA, is able to inhibit cancer cell growth by enhancing immune responses. Several *in vitro* studies have demonstrated that cancer cells transfected with PD-L1 siRNA are more sensitive to T cell killing compared to control groups (48, 49). The *in vivo* anticancer ability of PD-L1 siRNA has been further evaluated using the lymphoma solid tumor model. The knockdown of PD-L1 on cancer cells reduced tumor proliferation, tumor growth and cell cycle progression, and tumor invasion. Furthermore, PD-L1 knockdown reversed the resistance to chemical drug cisplatin, suggesting the role of PD-L1 in overcoming cancer drug resistance (50). Note that all these studies did not report the distribution of intracellular and extracellular PD-L1s but the outcomes indicate that PD-L1s play different roles in different cancer types. Although these studies did not directly compare the treatment efficacy of antibody blockade and gene silencing, we expect that gene silencing is superior in fully stopping mPD-L1 production and function.

Besides cancer cells, the expression of PD-L1 on the immune cell surface potentiates another anticancer strategy, i.e., improving T cell anti-tumor activity by suppressing PD-L1 on immune cells. The recent investigation highlights that the PD-L1 blockade influences the objective responses among PD-L1 negative patients (51, 52). PD-L1, as well as PD-L2 on antigen-presenting cells (APCs) can interact with PD-1 on CD 8^+^ T cells, resulting in down-modulation of T cell immune activity. Karwacz et al. utilized PD-L1 specific shRNA to suppress PD-L1 expression in bone-marrow derived dendritic cells (BMDCs) and demonstrated that the interference of PD-1/PD-L1 interaction led to down-modulation of TCR *via* casitas B-lymphoma (Cbl)-b E3 ubiquitin ligase upregulation in CD8+ T cells, and sequentially enhanced anti-tumor immune responses (53). Similarly, Hobo et al. reported that knockdown of PD-L1 and PD-L2 on monocyte-derived DCs strongly augmented T cell proliferation and cytokine production. The PD-L1 based gene therapy therefore improves the efficacy in cancer patients through modulated DCs (54–58).

### Epigenetic PD-L1 Suppression With miRs

**Figure 3:**
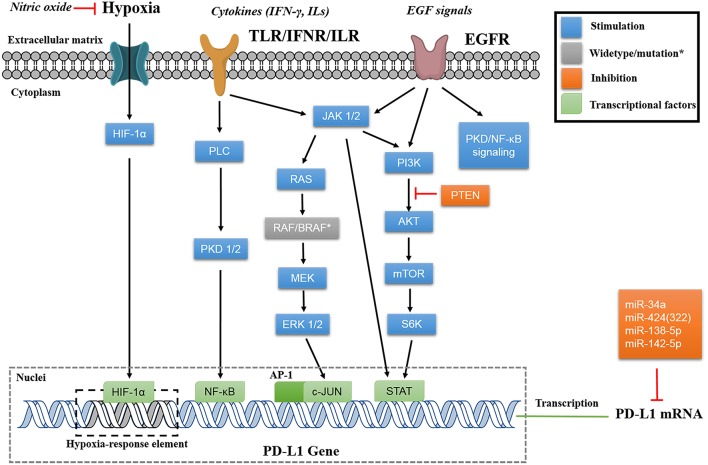
Signaling pathways of PD-L1 regulation. The instinct PD-L1 expression is regulated by translational factors (HIF-1α, NF-κB, AP-1, and STATs) that binds to the gene promoter. The extracellular signals (hypoxia, cytokines, and EGF signals) will be transduced *via* different pathways (mainly through MAPK or PI3K/AKT) to regulate PD-L1 expression on transcriptional level. Some miRs are able to bind to 3′-UTR of PD-L1 mRNA for post transcriptional regulation.

Apart from direct PD-L1 suppression using its siRNAs, the epigenetic regulation using several miRs also shows potential therapeutic efficacy by affecting PD-L1 expression. The miRs, such as miR-34a, miR-424(322), miR-138-5p, miR-142-5p, are able to bind directly to the 3′-UTR of PD-L1 mRNA, and downregulate PD-L1 expression (Figure 3) (59–62). Interestingly, the miRs would also affect the upstream regulatory pathway of PD-L1. For example, miR-200, known as a cell-autonomous suppressor of EMT (epithelial-to-mesenchymal transition) and metastasis, downregulates PD-L1 via abolishing the ZEB-1/miR-200 axis (63, 64). On the contrary, miR-20b, miR-21, and miR-130b positively affect PD-L1 expression in colorectal cancer through inhibiting the expression of PTEN, which abolishes the PI3K mediated PD-L1 upregulation (65). The positive relevance of miRs and PD-L1 expression makes PD-L1 a putative biomarker in the miR mediated pathway cascades (66).

### Limitation of Gene Silencing

The gene silencing method has proved to be efficient in “switching off” PD-L1 expression, resulting in low protein production at any location. Based on the *in vivo* results (50), gene silencing drugs, such as siRNA and miRNA, are effective in downregulating PD-L1 expression, suggesting that the endogenous molecular regulation would further affect the PD-L1 based therapy. The intracellular PD-L1 formats, nPD-L1 and cPD-L1, can directly respond to gene silencing, resulting in decreased levels of mPD-L1 due to self-metabolization and lack of endogenous supplements. The genetic modulation of PD-L1 in immune cells leads to hyperactive immune responses, including enhanced T cell proliferation, antigen-specific responses, and cytokine secretion. These reactions benefit the treatment for patients with both PD-L1 negative and positive cancers.

The main issue for gene silencing is delivery. The oligonucleotides are negatively charged, endowing them difficult to be internalized, and in particular, the RNAs (miR, shRNA, and siRNA) are vulnerable to RNase in the circulation. Therefore, suitable delivery systems are required for efficient transfection. Viral vector transfection methods are able to deliver genetic material to host cells with a high efficiency but their biosafety is a big concern. While non-viral transfection methods generally have a lower efficiency, there are considerably less safety concerns associated making them a hot topic in gene silencing research. Non-viral transfection methods utilize carriers such as calcium carbonate/phosphate (67, 68), inert gold nanoparticles (69), carbon/silicon based nanomaterials (70, 71), layered double hydroxide nanoparticles (72), various polymers (73), and positively charged lipids (74). The versatile nanoparticles provide custom- designed delivery platforms to different cases. In 2017, the first gene silencing based therapeutics, the liposomal miR-34a mimic, underwent Phase I clinical trials (59, 75). The development of gene silencing drugs has highlighted barriers such as efficient delivery, off-target effects, potential mutagenesis, and even some ethical arguments that need to be addressed to increase treatment efficiency (76). Thus, the majority of these gene silencing drugs are temporarily tested *in vitro*, or in mouse models. It has a long way to go from mice to non-human primates, which are ideal in this regard as they are more closer to human beings (77).

## Inhibition of PD-L1 Regulatory Pathways

### Regulation of PD-L1 Expression

PD-L1 expression is mainly regulated via MAPK (RAS/RAF/MEK/ERK) and PI3K/Akt pathways, and can be controlled by many intracellular and extracellular signals (Figure 3). Inhibiting these pathways can regulate PD-L1 expression, thus benefiting the cancer therapy. In addition, investigating PD-L1 regulatory pathways would potentially identify new inhibitors. Cataloged by the stimulation source, PD-L1 can be assorted as constitutive PD-L1 and inducible PD-L1, corresponding to innate and adaptive immune response, respectively (16). The constitutive PD-L1 expression is driven by endogenous oncogenic pathways, whereas the inducible PD-L1 expression is motivated by exogenous signals.

The upregulation of constitutive PD-L1 is strongly dependent on the activation of MAPK pathway, primarily of the kinases such as RAS, RAF, MEK, and ERK (4, 78). RAS/MEK pathway upregulates PD-L1 expression post-transcriptionally *via* TTP mediated increase of PD-L1 mRNA stability (79). The oncogenic activation of ERK 1/2 is proved to be involved in PD-L1 expression. The phosphorylation of these downstream oncogenes of MEK is able to upregulate PD-L1 expression transcriptionally (78, 80). In the study of BRAF inhibition-resistant melanoma, the researchers found that the depletion of JNK and ERK 1/2 synergistically suppresses PD-L1 expression. These findings support that c-JUN, an inducible transcription factor that can be modulated by JNK and ERK 1/2, plays an important role in regulating PD-L1 expression (4, 81). The mechanism of c-JUN based modulation might be that c-JUN, as the component of AP-1 transcription factor, binds to the enhancer element on PD-L1 gene and augments the transcription signal (82).

Another crucial modulation of constitutive PD-L1 expression is the PI3K/AKT/mTOR pathway. Moderate effects on PD-L1 expression can be achieved by downregulating either PI3K, AKT, or mTOR in glioma, NSCLC, breast, and prostate cancers (83–85). Activation of PI3K/AKT signaling pathway via other mechanisms, such as PTEN loss, increases PD-L1 expression as well (86). The mechanism of this PI3K/AKT/mTOR pathway may involve in its associated oncogenic activation of STATs (such as STAT 1/3). Among these STAT oncogenes, most investigations are focused on STAT3, which is believed to transcriptionally modulate PD-L1 expression as a part of the promoter, and exhibit the synergistic inhibition of PD-L1 expression with c-JUN in both carcinoma and APCs (4, 87, 88).

The inducible PD-L1 expression predominantly relies on extracellular signals, including cytokines, epidermal growth factors, and extracellular hypoxia conditions. The exogenous signal passes through certain cascade reactions that may crosslink to the triggered constitutive PD-L1 expression, and then affects the transcriptional factors to initiate PD-L1 encoding. A variety of cancer cell lines are cytokine inducible for PD-L1 upregulation as a rapid resistance to immune response (89–91). Among these, interferon-γ (IFN-γ) induced PD-L1 expression is well-studied. The MAPK and JAK-STAT-IFR signaling pathways are regulated by PKD1/2 activation and involved in this regulation (89, 92, 93). In addition, the activation of NF-κB is strongly associated with PD-L1 expression induced by IFN-γ and TNF-α (94). In particular, the IFN-γ induced PD-L1 expression on vemurafenib resistant cells is dependent on NF-κB, and not abolished by the inhibition of MAPK or PI3K/AKT pathways (95). A study also reported rapid PD-L1 upregulation responding to the NK supernatant with IFN-γ secretion, and this modulation seems to be related to JAK1/2-STAT1, without activation of other STATs, ERK, or AKT (96).

Similarly, PD-L1 expression is remodeled by EGF post-transcriptionally. PD-L1 overexpression in resected NSCLC tissue samples is positively correlated with EGFR expression while inversely correlated with HER2 expression (97). The potential mechanism involves in the enhancement of STAT3 signaling, including the augment of PI3K-AKT and/or IL6-JAK-STAT3 (97, 98). The activation of AKT by EGF signaling suppresses GSK3β activity through Ser9 phosphorylation, thus abolishing the phosphorylation-dependent proteasome degradation of PD-L1 (24). The activation of EGF signaling pathway upregulates PD-L1 expression via EGR-PLC-γ and ERK-MAPK as well (99). Similar to the regulatory PD-L1 expression induced by IFN-γ, the oncogenic EGF signaling pathway is another PD-L1 based cancer immune escape mechanism (100).

Recent studies have highlighted the role of extracellular hypoxia on PD-L1 suppression, as regulated by the intracellular HIFs. For instance, the nitric oxide (NO) signaling is able to block HIF-1α accumulation in hypoxic cells, and sequentially prevent hypoxia-induced PD-L1 expression and diminish immune resistance (101). The inducible HIFs bind to the hypoxia-response element in the PD-L1 proximal promoter, and modulate its expression as transcription factors (102). HIF blockade has been shown to be efficient to suppress PD-L1 expression in a variety of cells, including oral squamous cell carcinoma, adenocarcinoma, and MDSCs (102–104).

### Chemical Inhibitors of PD-L1

Apart from antibody and siRNA approaches, commercialized chemical inhibitors can still benefit PD-L1 based treatments by regulating the relevant expression pathways. The elucidation of PD-L1 regulation mechanisms provides appealing therapeutic regimens in overcoming the related cancer immune resistance. Based on this knowledge, the upstream regulatory molecules are ideal targets for screening inhibitors. Note that the inhibitors mentioned here are only small chemical molecules, the majority form of regulatory inhibitors. Small molecule inhibitors competitively bind to the enzyme/receptor, and can be as effective as other form of inhibitors, such as antibodies and oligonucleotides. A variety of marketed chemical inhibitors are able to downregulate cellular PD-L1 expression, directly targeting regulatory signaling pathways associated with inducible PD-L1. In addition, the sPD-L1 can be reduced in expression treated with matrix metalloproteinase inhibitor (MMPI) (27).

Another possible inhibitor vemurafenib, a commercialized competitive enzyme inhibitor, is approved by FDA for the treatment of late stage melanoma with BRAF V600E mutation (site shown in Figure 2). The description of multiple resistance mechanisms, including reactivation of MAPK signaling via upstream RAS mutation or downstream MEK mutation, and alternative growth pathway such as PI3K signaling, has been done in clinical trials in order to circumvent resistance (105).

With PD-L1 regulatory mechanism being elucidated, the development of small molecule inhibitors sheds light on precise knockdown of aberrant oncogene expression. Compared to traditional chemotherapy, chemical inhibitors are more effective and less harmful to normal cells, with much clearer patient subset (4, 106). Like gene silencing method, the usage of chemical inhibitors can “turn off” the PD-L1 expression “pipeline.” Thus, the influence of chemical inhibitors on different PD-L1 formats is similar to that of gene silencing, with a potentially slower response depending on the target relevance to PD-L1 expression.

Compared to direct PD-L1 blockade/silencing, inhibition of its signaling network may facilitate the therapeutic effect via (1) obstruction of cellular biological functions, and (2) abolishing drug resistance. The PD-L1 modulation involves MAPK and PI3K/AKT pathways, which participate in regulating other biological responses such as proliferation and survival. Notably, the suppression of AKT would upregulate Caspase cascade and induce apoptosis. The suppression of PKD2, which regulates IFN-γ induced PD-L1 expression, is able to abolish P-glycoprotein associated multidrug resistance (MDR). Taken together, the inhibition of signal pathways may result in chemo-immune combination therapy with much better efficacy.

The deficiency of signaling pathway inhibition is that it may take time to reduce PD-L1 expression (31). Therefore, this approach may be not as sufficient as antibody at a very early stage, while still sufficient to regulate PD-L1 at the later stage. In order to overcome this deficiency and take the inherent advantage of small molecule inhibitor drugs, efforts have been made to directly target the immune checkpoint protein itself. At least 19 compounds are investigated, while all these examinations are now at the preclinical stage (3). No report claims any defined influence of these compounds on PD-L1 distribution, while we can reasonably postulate their characters from the knowledge of other chemical inhibitors. Due to its small molecule size compared antibodies, the internalization of these compounds may be easier, while the elimination may be much quicker (clearance of chemical inhibitor Cobimetinib is 331 L/day, while that of PD-L1 antibody durvalumab is only 0.2 L/day). So these potential PD-L1 inhibitors may be easier to control intracellular PD-L1 formats. As for more mPD-L1, either higher dose or frequent administration of inhibitors is required.

As it stands direct comparison between antibody blockage and chemical inhibition is not viable due to lack of good evidence. However, just from the view of total inhibition of PD-L1 production and function, chemical inhibition may be more effective although the side effects of chemical inhibitors could be more significant than antibodies.

## Future Prospect for PD-L1 Based Cancer Combinational Therapy

Based on the above review, it seems that none of the current three treatment methods is a perfect regime to adequately inhibit PD-L1 production and function for effective immunotherapy. We think that it is important to consider controlling intracellular PD-L1 formats in future immunotherapy. The intracellular PD-L1 formats will not be affected if the treatment utilizes only antibody to block the membrane format. However, for PD-L1 positive cancer cells, (of a patient) antibody blockade will be the most effective and direct way to restore T cell immunity. Therefore, we believe the future treatment strategy will probably combine these methods as a combinational therapy.

Aiming to modulate PD-L1 expression in a more effective way, and taking the benefits from other aspects such as inhibition of cell proliferation and basic metabolism, the exploration of combinational treatments has been performed in many late stage cancer patients, and leads to improved clinical responses (107). The current combination therapies can be divided into three types: (1) antibody blockade + chemical inhibition, (2) antibody blockade + gene knockdown, and (3) gene knockdown + chemical inhibition. Thanks to the commercialized products, the first two types involving PD-L1 antibody attract more attention. The monotherapy using marketed PD-L1 antibodies can be boosted by either target therapy using chemical inhibitors or gene knockdown. According to the data from Clinicaltrials.gov, 16 clinical trials (14 under recruiting) using combination therapies with Atezolizumab and the chemical inhibitors were submitted in 2017 (Table 4). With the involvement of chemical inhibitors, the applicable cancer type is much broader than that of PD-L1 antibody monotherapy. Meanwhile, combination of PD-L1 and its certain upstream regulation inhibitors may exert synergy that facilitates the therapy, as indicated by their serial (or partially parallel mixed) network topology structure (108). For instance, Cobimetinib, a marketed MEK inhibitor, was used in 12 of these trials, and the combination regimens resulted in longer progression-free survival than monotherapy using PD-L1 antibody or Cobimetinib in treating colorectal cancer and melanoma (109, 110). In the meantime, the therapies combined with gene silencing are limited to *ex vivo* stage due to the lack of marketed gene drugs, which might be a new research hot-spot in the future. Consideration of thorough “knock-out” of PD-L1 in genome using CRISPR technology will also be important as a new approach, and related investigations are undergoing (111).

**Table 4:** Combination therapies involving PD-L1 antibody (Atezolizumab) and the regulatory inhibitors in 2017.

**NCT number**	**Recruitment**	**Conditions**	**Other interventions**	**Phase**
NCT03434379	Recruiting	Carcinoma, hepatocellular	Bevacizumab, Sorafenib	III
NCT03395899	Recruiting	Breast cancer Estrogen receptor-positive breast cancer	Cobimetinib, Ipatasertib, Bevacizumab	II
NCT03363867	Not yet recruiting	Ovarian cancer, fallopian tube cancer, primary peritoneal carcinoma	Bevacizumab, Cobimetinib	II
NCT03340558	Not yet recruiting	Metastatic colorectal cancer	Cobimetinib	II
NCT03337698	Recruiting	Carcinoma, NSCLC	Cobimetinib, RO6958688, Docetaxel, BL-8040, Tazemetostat, CPI-444, Pemetrexed, Carboplatin, Gemcitabine	I/II
NCT03312630	Recruiting	Multiple myeloma	Cobimetinib, Venetoclax	I/II
NCT03292172	Recruiting	Advanced ovarian cancer, triple negative breast cancer	RO6870810	I
NCT03280563	Recruiting	Breast neoplasms	Bevacizumab, Cobimetinib, Exemestane, Fulvestrant, Ipatasertib, Tamoxifen	I/II
NCT03273153	Recruiting	Advanced BRAFV600 wild-type melanoma	Cobimetinib, Pembrolizumab	III
NCT03264066	Recruiting	Solid Tumors	Cobimetinib	II
NCT03202316	Recruiting	Malignant neoplasm of breast	Cobimetinib, Eribulin	II
NCT03201458	Recruiting	Non-resectable cholangiocarcinoma	Cobimetinib, Laboratory biomarker analysis	II
NCT03178851	Recruiting	Malignant melanoma	Cobimetinib	I
NCT03170960	Recruiting	Urothelial carcinoma, renal cell carcinoma	Cabozantinib	I/II
NCT02314481	Recruiting	Malignant neoplasms of digestive organs, melanoma, other malignant neoplasms of skin, appendiceal adenocarcinoma, cutaneous squamous cell carcinoma, small bowel adenocarcinoma	Cobimetinib	II
NCT02314481	Recruiting	NSCLC	Vemurafenib, Alectinib, Trastuzumab emtansine	II

**Figure 4:**
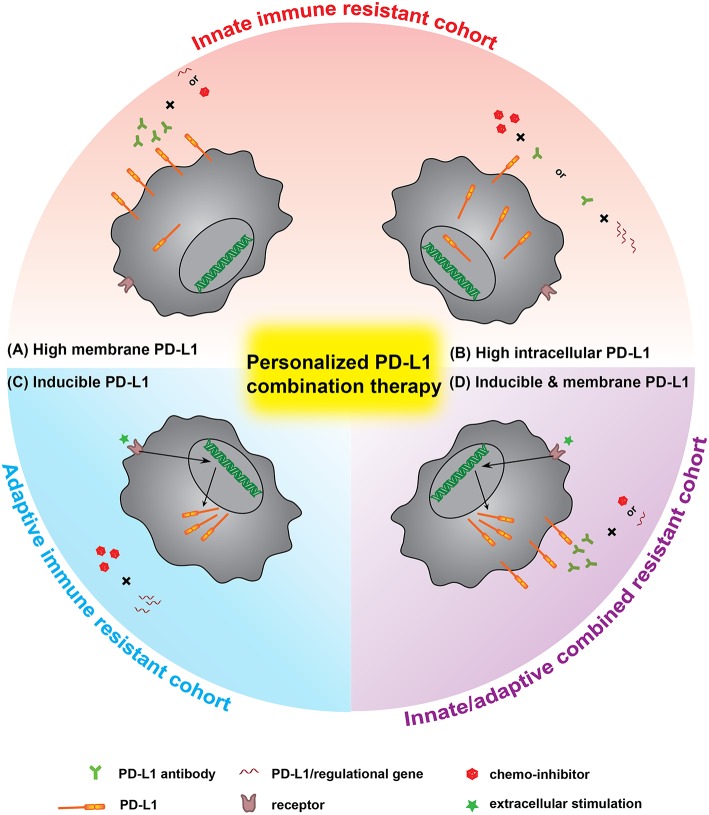
The personalized PD-L1 combination therapy, a prospect of optimal PD-L1 immunotherapy guided by PD-L1 distribution and immune resistance of patients. Innate immune resistant cohort: **(A)** for cells with high constitutive mPD-L1, the regimen would mainly rely on antibody blockade while the combination of gene silencing or chemical inhibitor would benefit the treatment. **(B)** For cells with high intracellular PD-L1, the regimen should more rely on gene knockdown or inhibition method, supported by PD-L1 antibody. Adaptive immune resistant cohort: **(C)** the inducible PD-L1 is much easier to be controlled by gene silencing and chemical inhibitor combination therapy. Combined immune resistance cohort: **(D)** for cells with both high constitutive and inducible PD-L1, the regimen would be better to choose the combination of antibody + gene silencing or antibody + chemical inhibitor.

With the development of PD-L1 based cancer therapy agents, the future combination therapy will be personalized, guided by the characteristics of patients' cancers based on: (1) the information of PD-L1 distribution in cancer cells, and (2) PD-L1 mediated innate/adaptive immune resistance. This postulation that how PD-L1 distribution and adaption in individual patient guides the choice of an optimal combination regimen is illustrated in Figure 4. The innate immune resistance will firstly be checked. For those cancers highly expressing mPD-L1, such as melanoma and NSCLC, the ideal regimen would be based on antibody blockade, in combination with chemical inhibition or gene knockdown for enhanced efficiency (112). Whereas, for cancers with high intracellular PD-L1 expression that do not show significant innate immune resistance, a possible regimen focusing on using chemical inhibitors or silencing gene with supporting therapy by PD-L1 antibody could be taken into consideration, especially in some extreme situations like multidrug-resistance patients with the high intracellular PD-L1. For cancers with low constitutive PD-L1 expression, the PD-L1 antibodies may also benefit the therapy. Indeed, most pilot therapies, such as radiation or chemotherapy, lead to cancer acquisition of immune resistance (113). The stimulation of certain pro-anticancer cytokines (e.g., IFN-γ) upregulates PD-L1 expression. The following therapy inhibiting PD-L1 expression could reverse the adaptive immune resistance. For the cohort with innate/adaptive combined resistance, the use of combinational methods to knockdown/inhibit the inducible molecular pathway would be a more efficient way. When combined with PD-L1 antibody, it would further block the possible surface mPD-L1 to enhance the treatment. We have observed this phenomenon in both mPD-L1 high cell line (B16F0) and mPD-L1 low cell line (MCF-7 and 4T1, unpublished data).

The current clinical trials of PD-L1 antibody broaden the applicable range with other therapies and enhance the therapeutic efficacy. The clinical trials documented in Clinicaltrials.gov from January to May 2019 on PD-L1 antibody-based combination therapies are listed in Table 5. Although most of the trials are endeavored in optimizing the conventional therapies such as radiation- and chemotherapy, some trials are aimed to develop regimens with immunoregulators and pathway/receptor inhibitors. Remarkable clinical outcomes were achieved by combination of CTLA-4 and PD-1/PD-L1 antibodies, providing ORR > 50% in certain cancer cases (114, 115). Regarding to the adaptive PD-L1 immune resistance, the investigations of VEGFR and EGFR inhibitors in combination with PD-L1 antibodies are worth noticing due to the enhanced antitumor activity by their considerable synergistic effects (116, 117). With the deep understanding of PD-L1 distribution and its influence on therapeutic options, more profitable outcomes can be expected in the future.

**Table 5:** Application of PD-L1 antibodies in cancer combination therapy in 2019.

**PD-L1 Ab**	**Combination**	**Involved trial records**
Atezolizumab (Tecentriq)		**40 (Total)**
	Radiation	6
	Chemotherapy	6
	Regulatory inhibitors	2
	Receptor inhibitors	13
	Immunoregulator antibody (CD73)	1
	Immunoregulator antibody (PD-1)	2
	Immunoregulator antibody subtotal	7
	PARP inhibitor	3
Avelumab (Bavencio)		**21 (Total)**
	Radiation	5
	Chemotherapy	6
	Regulatory inhibitors	1
	Receptor inhibitors	5
	Immunoregulator antibody (IDO)	2
	Immunoregulator antibody subtotal	4
Durvalumab (Imfinzi)		**56 (Total)**
	Radiation	14
	Chemotherapy	18
	Regulatory inhibitors	8
	Receptor inhibitors	9
	Immunoregulator antibody (CTLA-4)	12
	Immunoregulator antibody (CD73)	6
	Immunoregulator antibody subtotal	24
	PARP inhibitor	3

## Conclusion

The PD-1/PD-L1 immune checkpoint interaction is an arch-important regulator in tumor immune escape. The blockade or downregulation of PD-L1 in both immune cells and tumor cells is beneficial to breaking down the negative immune regulation and evoking enhanced immunity against tumor. Emerging data on PD-L1 biology demonstrate its relevance to other behaviors of cancer cells such as drug resistance and metastasis, highlighting its multiple roles in cancer development, additionally to immune regulation. Current treatment strategies mainly focus on PD-L1 expressed on the cell membrane (mPD-L1), other formats of intracellular/extracellular PD-L1 are not well-studied and understood. Therefore, these should be an important direction of related research. The methods of gene knockdown and chemical inhibition of signaling pathways have been proved to be efficient in inhibiting intracellular PD-L1 production but not effective for serum PD-L1 format (sPD-L1). They may also be ineffective for PD-L1 already expressed on the cell surface. Based on these understandings, we postulate that the future direction of PD-L1 based cancer immunotherapy will lie in combined and personalized cancer therapies. Expectedly, the future combination approaches will strategically utilize antibody blockade, gene knockdown, and/or regulatory pathway inhibition based on patient PD-L1 distribution information, which will lead to more effective cancer immunotherapy.

## Author Contributions

YW reviewed the literature and wrote the main part of first draft. WC contributed to the antibody advances. ZX and WG devised the main conceptual idea and polished the draft. All authors contributed to revisions of the manuscript, and approved it for publication.

### Conflict of Interest Statement

The authors declare that the research was conducted in the absence of any commercial or financial relationships that could be construed as a potential conflict of interest.
